# *Pituranthos chloranthus* Oil as an Antioxidant-Based Adjuvant Therapy against Cisplatin-Induced Nephrotoxicity

**DOI:** 10.1155/2020/7054534

**Published:** 2020-05-14

**Authors:** Aida Lahmar, Zaineb Dhaouefi, Rihab Khlifi, Fairouz Sioud, Leila Chekir- Ghedira

**Affiliations:** ^1^Unit of Bioactive and Natural Substances and Biotechnology UR17ES49, Faculty of Dental Medicine, University of Monastir, Avicenna Street, Monastir 5000, Tunisia; ^2^Faculty of Pharmacy, University of Monastir, Avicenna Street, Monastir 5000, Tunisia; ^3^Faculty of Sciences of Tunis, University of Tunis El Manar, Tunis, Tunisia

## Abstract

The therapeutic outcome of cisplatin is limited due to its adverse side effects in normal tissues. Despite its potent antineoplastic effect, cisplatin is known by a relevant collateral action, for instance, acute renal failure. The aim of this study was to assess the effectiveness of *Pituranthos chloranthus* (PC) essential oil for contracting cisplatin-induced toxicity, in Balb/c mice. The standard mouse model of cisplatin-induced acute kidney injury (AKI), consisting of one intraperitoneal injection of cisplatin (20 mg/kg), was adopted. Mice were pretreated by intraperitoneal administration of PC (5 and 10 mg/Kg b.w) for one week. Cisplatin induced alteration in renal and liver functions, evidenced by increased serum biomarkers levels (creatinine, ALT, and AST). Significant mitigation of cisplatin-induced toxicity was confirmed by lowered levels of serum biomarkers and reduced DNA damage in liver and kidney. PC also restored the alterations in oxidative stress markers and proinflammatory cytokine IFN-*γ* level. Overall, this study provides, for the first time, that PC can be applied as an antioxidant-adjuvant treatment to mitigate cisplatin-induced renal failure.

## 1. Introduction

Cisplatin is one of the options that is gaining interest in clinical oncology, since it has shown effectiveness against various types of cancers [1]. However, several reports revealed its serious side effects including nephrotoxicity, ototoxicity, myelosuppression, and gastrotoxicity. In particular, cisplatin-induced nephrotoxicity can cause an acute kidney injury (AKI), occurring in 20–30% of patients [2]. The nephrotoxicity is manifested mainly by damages in the outer stripe of the outer medulla of the proximal tubule [3]. The mechanism of cisplatin-induced nephrotoxicity is a complex process involving oxidative stress, genotoxicity, and inflammation. Cisplatin concentrate in the renal tubular epithelial cells to levels five times higher than those in blood, inducing, hence, the cell death in renal cells [4]. Furthermore, DNA-binding activity of cisplatin is not specific and may adversely affect several nontarget cells [5]. Some of suggested strategies have been implemented to diminish or prevent nephrotoxicity of cisplatin. The standard approach for prevention of cisplatin-induced nephrotoxicity is the administration of lower doses of cisplatin in combination with full intravenous hydration prior and after cisplatin administration [6]. Cisplatin-induced oxidative stress in the kidney may be prevented by natural antioxidant compounds. Amifostine, a cytoprotective adjuvant, is the only FDA approved therapeutic agent for the reduction of cisplatin-induced renal toxicity [7]. Nevertheless, the application of amifostine is limited due to its side effects, cost, and concerns about possible interference with the antitumor activity of cisplatin [8]. On the contrary, natural antioxidants have been known to impart protection without compromising the antitumor potential of chemotherapeutic drugs and side effects Therefore, studies on screening of potential phytochemicals to enhance the efficacy of chemotherapeutic agents and reducing its undesirable side effects are of great interest. The north endemic plant, *Pituranthos chloranthus* (PC), locally named Guezzah, is a small aromatic plant. Several research works were conducted on *Pituranthos chloranthus* essential oils, in particular, on its phytochemical content, antimutagenic activities [9], and antimicrobial potential [10]. Hence, the present investigation has been carried out to study the possible protective effects of pretreatment with *Pituranthos chloranthus* on the genotoxicity, oxidative stress, and inflammation, induced by cisplatin, *in vivo*.

## 2. Materials and Methods

### 2.1. Chemical Agents

Cisplatin (CP) was purchased from local pharmacies under the trade name Cisplatin Mylan (Oncotec Pharma Produktion GmbH, Germany) and administered intraperitoneally.

### 2.2. Animal Handling and Care

Healthy and pathogen-free male BALB/c mice (20–22 g) were obtained from Pasteur Institute (Tunis, Tunisia). The mice were fed a commercial pellet diet and water *ad libitum* during the experiment period. All experiments were performed in accordance with the guidelines for the care and use of laboratory animals as published by the National Institute of Health.

### 2.3. Dose Selection

The standard mouse model of cisplatin-induced AKI, consisting of one intraperitoneal injection of cisplatin (20 mg/kg), was adopted [11]. Prior to cisplatin injection, mice were pretreated by different doses of PC (5 or 10 mg/kg b.w.) and evaluated for possible mitigation of cisplatin-induced toxicity.

Thirty-six mice were randomly assigned to six groups (six animals per group):

- GROUP 1 (vehicle): mice were injected intraperitoneally (i.p.) with PBS daily for 10 days, without any drug treatment.
- GROUP 2 (PC 5 mg/kg): mice were injected i.p. with PC 5 mg/kg b.w. daily for 10 days.
- GROUP 3 (PC 10 mg/kg): mice injected i.p. with PC10 mg/kg b.w. daily for 10 days.
- GROUP 4 (cisplatin control): mice were injected i.p. with PBS daily for 10 days. On the 7th day, a single dose of cisplatin (20 mg/kg, i.p.) was administrated to induce renal injury.
- GROUP 5: mice were injected i.p. with PC 5 mg/kg b.w. daily. On the 7th day, a single dose of cisplatin (20 mg/kg, i.p.) was given. Then, PC administration was continued up to day 10.
- GROUP 6: mice were injected i.p. with PC 10 mg/kg b.w. daily. On the 7th day, a single dose of cisplatin (20 mg/kg, i.p.) was administrated. Then, PC administration was continued up to day 10.

Mice were sacrificed 72 h days after cisplatin injection. Blood samples were collected into EDTA vacutainers to perform biochemical analyses. Comet assay and different tests were conducted in the same set of animals.

### 2.4. Change in Bodyweight and Somatic Index

The bodyweight of each mouse was recorded in the end of experiment, and the percentage change in bodyweight was subsequently determined. After mice were sacrificed, kidneys and spleens from the animals were removed, weighed, and the ratio between organ weight and bodyweight of the animals was calculated. Data of somatic index are expressed as a ratio of organ weight over bodyweight.

### 2.5. Liver Function Test (LFT) and Renal Function Test (RFT)

For the assessment of LFT, changes in the activity of serum alanine aminotransferase (ALT) and aspartate aminotransferase (AST) were assessed. Creatinine levels were determined to evaluate the renal function (RFT). The measurement of these biochemical parameters was done in the clinical chemistry analyzer Cx72 (COBAS).

### 2.6. Genotoxicity Assay

The alkaline comet assay was carried out as described by [12] with minor modifications. Cellular suspension obtained from each organ was included in an agarose gel and subjected to lysis followed by electrophoresis. The total score of DNA damage was calculated by the following equation.

Total DNA damage = percentage of cells in class (0) *x*0 + percentage of cells in class (1) *x*1 + percentage of cells in class (2) *x*2 percentage of cells in class (3) *x*3 percentage of cells in class (4) *x*4.

### 2.7. ELISA

Serum tumor necrosis factor-alpha (TNF- *α*) levels were determined using the Quantikine ELISA kit (Biovendor research and diagnostic products) according to the manufacturer's protocol.

### 2.8. Biochemical Markers of Oxidative Stress

Kidney and liver tissues were homogenized with 10 volumes of ice-cold saline PBS and were centrifuged at 4,000 rpm for 15 min at 4 °C, and the supernatant was stored at −80 °C until used for the determination of malondialdehyde levels, catalase activity, and SOD activity.

### 2.9. Determination of Malondialdehyde (MDA) Level

Lipid peroxidation was evaluated by monitoring the formation of malondialdehyde (MDA) level in renal and liver extracts, using the method described by [13] with modification. Briefly, 50 *μ*l of the homogenate was mixed with trichloroacetic acid (1 mL) and thiobarbituric acid (1 mL). The reaction mixture was heated (60 min. at 95 °C) and then cooled at room temperature for one hour. After cooling, the absorbance of the pink color was measured at 532 nm. The levels are expressed as nmol/mg proteins.

### 2.10. Analysis of Superoxide Dismutase Activity

The superoxide dismutase (SOD) activity was determined in the extracts of different organs (the kidney and liver). Nitroblue tetrazolium (NBT) reduction is used as an indicator of SOD activity [14]. The reaction mixture consisted of 50 *μ*l of the liver or kidney, 2 mM NBT, 10 mM methionine, 2.4 mM riboflavin, and 0.1 mM EDTA in a final volume of 1.7 mL. The reaction was carried out for 15 min and illuminated with a UV fluorescent lamp. The absorbance was then measured at 560 nm. An enzymatic unit was defined as the amount of cytosol required to inhibit 50% of the reaction without enzyme.

### 2.11. Measurement of Reactive Oxygen Species Level in Bone Marrow Cells

The bone marrow from the femurs of animals was flushed out with PBS into a centrifuge tube. The cells were collected by centrifugation at 1500 rpm for 10 min. Cell pellets were resuspended with 1× PBS. Intracellular ROS production was measured with the help of oxidative fluorescent probe DCFH-DA. Cells (5 × 10^5^) were stained with 10 *μ*M DCFH-DA and incubated in the dark for 30 min to allow the formation of fluorescent dichlorofluorescein (DCF) and then analyzed using a fluorescence microplate reader (Biotek, Winooski, USA), with 538 nm emission and at 485 nm excitation filters [15].

### 2.12. Statistical Analysis

Data obtained from analyses at each parameter were evaluated by one-way analysis of variance (ANOVA). The general linear model with repeated measures was used to different parameters. Tukey's test was used for multiple comparisons of the means. The significance level was set at *p* < 0.05. SPSS (v. 18.0) software was used to carry out the statistic test.

## 3. Results

### 3.1. PC Mitigate Cisplatin-Induced Bodyweight Loss and Organosomatic Index Changes

**Figure 1:**
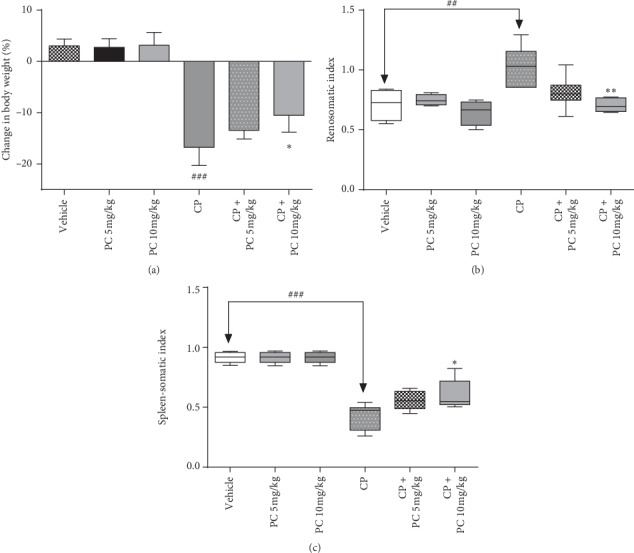
Effect of PC on cisplatin-induced variation in bodyweight and the renosomatic index. Results are expressed as mean ± SD (*n* = 6).^.^^*∗*^*p* < 0.05 and ^*∗∗*^*p* < 0.01 mean significant difference between treated animals and the cisplatin-treated group (CP). ^##^*p* < 0.01 and ^###^*p* < 0.001 mean significant difference between vehicle and the cisplatin-treated group (CP).

To determine the effect of cisplatin administration on bodyweight of animals, we monitored the bodyweight changes, at the end of the experiment. Vehicle and PC-treated mice have gained weight (Figure 1(a)). However, cisplatin injection led to a dramatic loss in the average bodyweight, by 16 %. To confirm the harmful effect of cisplatin on the kidney and spleen and also to investigate whether PC exerts any protective effect on these organs, the organosomatic index was determined at the end of the experiment. An increase in the organosomatic index of the kidney in the cisplatin-treated group was recorded. Nevertheless, exposure to cisplatin reduced spleen-somatic index ratio. The results obtained show that the administration of PC at a dose of 10 mg/Kg bodyweight mitigates this abnormality (Figures 1(b) and 1(c)).

### 3.2. PC Attenuate Genotoxic Effect of Cisplatin in Liver and Renal Cells

**Figure 2:**
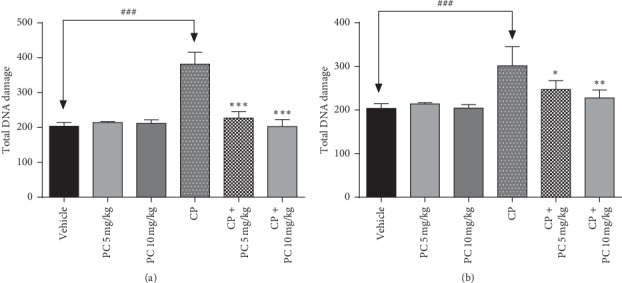
Inhibitory effect of PC on the genotoxicity induced by cisplatin in liver cells (a) and in kidney cells (b) isolated from Balb/c mice using the alkaline comet assay. Values are expressed as mean ± SD (*n* = 6). ^*∗*^*p* < 0.05, ^*∗∗*^*p* < 0.01, and ^*∗∗∗*^*p* < 0.001 mean significant difference between treated animals and the cisplatin-treated group (CP). ^###^*p* < 0.001 means significant difference between vehicle and the cisplatin-treated group (CP).

The prevention of genotoxicity induced by cisplatin in renal and liver cells was carried out through the alkaline comet assay. Mice treated with cisplatin showed significant DNA damage in both renal and liver cells, as demonstrated by an increase in the percentage of total DNA damage (Figure 2). Animals treated with PC showed significant decrease in mean DNA damage.

The genotoxic potential of cisplatin has been linked to its ability to cross link with the purine bases on the DNA, causing, hence, DNA damage in malignant cells [16]. However, this action is not tumor-specific and may affect normal cells.

### 3.3. Protective Effect of PC on Renal Function Test (RFT) and Liver Function Test (LFT)

Cisplatin is a potent nephrotoxic drug. Kidney function was impaired by cisplatin treatment as demonstrated by an increase in serum creatinine levels. This biomarker was significantly increased by 62% in the cisplatin-treated group. However, treatment of animals with PC restored the serum level of creatinine to a normal value (Table 1).

The alanine aminotransferase (ALT) and the aspartate aminotransferase (AST) levels were measured to monitor the hepatoxicity in the hosts. Significant increase in ALT and in AST activities were recorded after cisplatin administration alone. However, as compared to the cisplatin-treated group, concomitant treatment significantly mitigates ALT and AST activities. This effect appeared to be dose related.

### 3.4. PC Restores the Level of the Proinflammatory Cytokine IFN-*γ*

**Figure 3:**
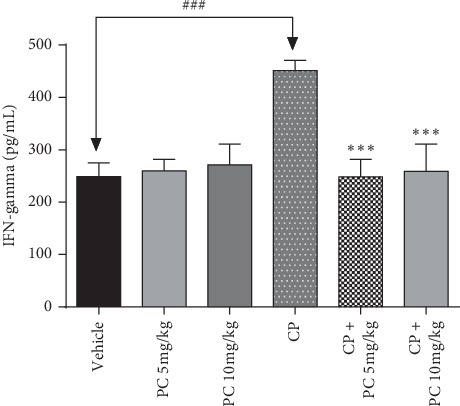
Effect of PC on cisplatin-induced variation in proinflammatory cytokine IFN-*γ* level. ^*∗∗∗*^*p* < 0.001 means significant difference between treated animals and the cisplatin-treated group (CP). ^###^*p* < 0.01 means significant difference between vehicle and the cisplatin-treated group (CP).

Interferon-gamma (IFN-*γ*) is a cytokine that plays a crucial role in cellular stress and cellular stress-related pathophysiology [17]. Quantification of IFN-*γ* was performed to study the inflammatory response in the cisplatin-treated group and to elucidate the plausible protective role of PC. Cisplatin induces an acute inflammatory reaction in mice, as revealed by the IFN-*γ* level. PC pretreatment significantly attenuated the increase in levels of IFN-*γ* (*p* < 0.001) as compared to the cisplatin control group (Figure 3).

### 3.5. PC Ameliorates the Free Radical Status

To substantiate whether ROS generation is responsible for the pathophysiology induced by cisplatin, DCF staining was performed. ROS production was fluorimetrically analyzed in mouse bone marrow cells. The excessive ROS production is evident in cisplatin-administered animals that correspond to a large number of superoxide anions generation.

**Figure 4:**
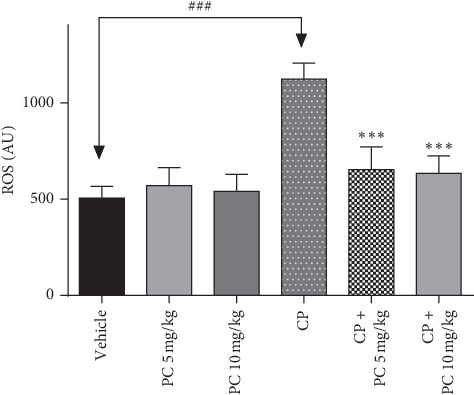
Effect of cisplatin and PC on intracellular ROS production in bone marrow cells isolated from Balb/c mice. ROS production was estimated by DCF staining. Results are expressed as mean ± SD (*n* = 6). ^*∗∗∗*^*p* < 0.001 means significant difference between treated animals and the cisplatin-treated group (CP). ^###^*p* < 0.01 means significant difference between vehicle and the cisplatin-treated group (CP).

Treatment with PC resulted in significant reduction of ROS production as is evident from (Figure 4). PC alone in different doses did not contribute to ROS accumulation and fluorescence intensity was found to be comparable to that of nontreated animals.

### 3.6. Effect of PC Treatment on Oxidative-Stress Parameters

**Figure 5:**
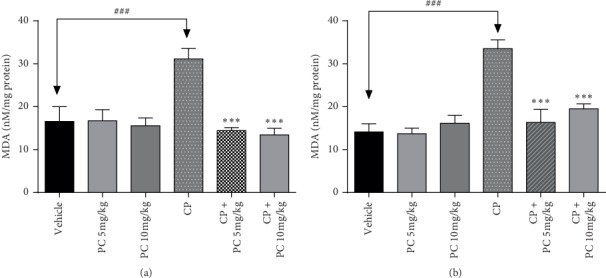
Lipid peroxidation as determined by MDA (malondialdehyde) levels in liver (a) and kidney (b) homogenates. Results are mean ± SD (*n* = 6). The statistical significance of results was evaluated two-way ANOVA followed by Tukey's multiple comparison test. ^*∗∗∗*^*p* < 0.001 means significant difference between treated animals and the cisplatin-treated group (CP). ^###^*p* < 0.01 means significant difference between vehicle and the cisplatin-treated group (CP).

**Figure 6:**
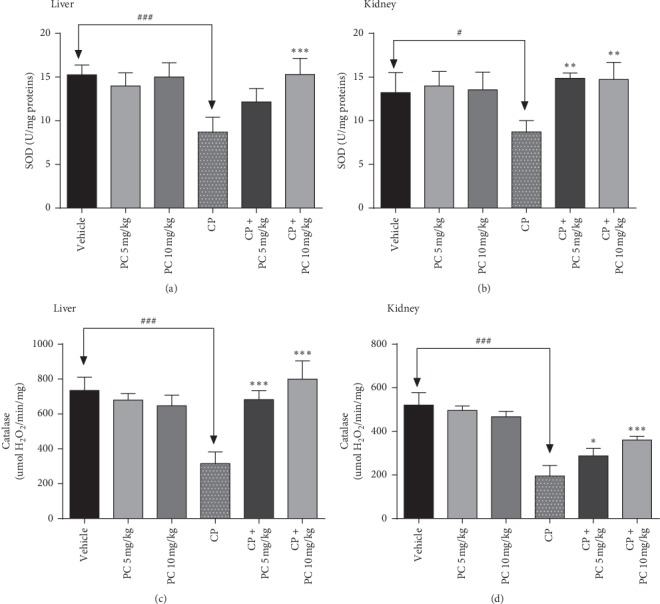
Superoxide dismutase (SOD) and catalase activity in the liver and kidney homogenates from mice treated by PC. Results are mean ± SD (*n* = 6). ^*∗*^*p* < 0.05, ^*∗∗*^*p* < 0.01, and ^*∗∗∗*^*p* < 0.001 mean significant difference between treated animals and the cisplatin-treated group (CP). ^#^*p* < 0.05 and ^###^*p* < 0.01 means significant difference between vehicle and the cisplatin-treated group (CP).

Malondialdehyde (MDA) is one of the final outputs of polyunsaturated fatty acid peroxidation in the cells, which considered as a substantial marker of lipid peroxidation [18]. Otherwise, superoxide dismutase (SOD) and catalase are enzymes that protect cells from radical attack [19]. In the current study, cisplatin-challenged mice showed a significant (*p* < 0.001) increase in the levels of MDA and a significant reduction in the activity of antioxidants SOD and catalase (*p* < 0.001), as compared to the normal group. PC intervention led to significant decrease in lipid peroxidation at the tested doses (Figures 5 and 6). Furthermore, animals pretreated with PC (5 and 10 mg/kg b.w.) showed a significant increase of the levels of SOD and catalase, in liver and kidney cells compared to the cisplatin-treated group.

## 4. Discussion

Clinical oncology has been increasingly confronted with the physiological side effects of cisplatin, despite its success against many forms of cancers [5]. The drug is not target-specific and causes genotoxic effects on normal cells, particularly the proliferative ones. Given the proven nephrotoxic effect of cisplatin, the alleviation of its oxidative damage to normal tissues is an issue to be covered. Combinatorial strategies of chemotherapy drugs and natural agents have developed in order to reduce the side effects of cisplatin [20]. The growing interest in natural antioxidants has challenged the scientific community to innovate concomitant diet along with conventional cancer therapies.

The aim of this study was to assess the potential of *Pituranthos chloranthus* essential oil to reverse the deterioration in a cisplatin-induced AKI model [21]. Cisplatin treatment caused a severe drop in bodyweight of mice, which could be explained by reduced appetite, dehydration, digestive disorders, and renal tubular injury [23].

Furthermore, the renosomatic index and spleen-somatic index were altered, in the cisplatin-treated group. The acute renal edema causes the increased renosomatic index, whereas the spleen-somatic index is reduced by apoptosis in splenic cells [22]. PC treatment, however, has been shown to be effective against these abnormalities. Cisplatin-induced AKI is characterized by an intense decrease in renal function, evidenced by increased serum creatinine levels. Furthermore, liver dysfunction was demonstrated by an increase in serum biomarkers ALT and AST.

The high genotoxic potency of cisplatin may be responsible for secondary malignancies, which have been observed in cured patients treated with cisplatin [24]. In the current study, this mutagenic potential was proved after cisplatin treatment in renal and liver cells, supporting earlier evidences of its genotoxic properties. Our data show that there was a significant increase in the total DNA damage score in the CP-treated group compared to the control group, whereas PC-treated animals revealed a reduced score, in liver and renal cells. A strong correlation was demonstrated between genotoxicity and oxidative stress status. Reactive oxygen species (ROS) trigger cell components, including DNA, and destroy their structure. Consequently, ROS is involved in the pathogenesis of cisplatin-induced kidney injury [25]. Consistent with previous evidences, our present study shows that cisplatin administration inhibited the activities of antioxidant enzymes SOD and catalase and led to an increase in MDA levels. Simultaneously, PC treatment restored the SOD and catalase activities and decreased significantly the amount of MDA, in liver and renal cells. The imbalance in antioxidant status can be explained by the excessive accumulation of ROS. Hydrogen peroxide and superoxide radicals contribute to cisplatin-induced toxicity. As evidenced from DCFH-DA staining in the bone marrow cells, cisplatin exposure caused ROS generation in the bone marrow cells and PC could mitigate that accumulation.

Several evidences support the role of ROS as a mediator of inflammation [26]. The proinflammatory cytokine IFN-*γ* g is a pleiotropic cytokine that is involved in various pathologies including kidney injury [27]. Kimura al.2012 reported that IFN-*γ* expression was enhanced following cisplatin injection. Our data revealed increased level of serum cytokine IFN-*γ* in experimental mice exposed to cisplatin, and treatment with PC succeeded to hamper this abnormality. An essential oil from selected plant counteracts the pro-oxidant status created by cisplatin administration, protecting, hence, treated animals. It is well known that plant essential oils with antioxidant potential have gained interest as adjuvant therapies in drug treatment. Antioxidant properties of essential oils are difficult to correlate to specific compounds because of their complexity and variability [6]. However, the antioxidant properties of the present essential oils could be attributed to specific components such as sabinene and limonene with proven antiradical potential [28]. Our previous study demonstrated the efficiency of PC to attenuate the potential pro-oxidant action of high oxygen atmosphere in red meat In this regard, the tested essential oil had been nominated as suitable candidates to guarantee food conservation.

## 5. Conclusion

PC treatment prior to cisplatin administration in mice can minimize cisplatin-induced nephrotoxicity, genotoxicity, and inflammation through enhancing oxidative status. The current finding suggests the efficiency of PC as a potential agent for the development of cisplatin chemotherapy adjuvant treatments.

## Figures and Tables

**Table 1:** Effect of PC on serum parameters ALT, AST, and creatinine levels, in cisplatin-treated mice.

	ALT (U/L)	AST (U/L)	Creatinine (µmol/L)
Vehicle	142,75 ± 20,0	49,33 ± 8,1	26 ± 10,5
CP	348 ± 22,5^###^	112 ± 6,9^##^	42 ± 10,9^##^
CP + PC 5 mg/kg	319 ± 46,5	69 ± 2,7	27 ± 1,4^*∗∗*^
CP + PC 10 mg/kg	271,5 ± 23,2^*∗*^	59 ± 14,3^*∗∗*^	28 ± 2,4^*∗∗*^

Results are mean ± SD (*n* = 6). ^*∗*^*p* < 0.05 and ^*∗∗*^*p* < 0.01 mean significant difference between treated animals and the cisplatin-treated group (CP).^##^*p* < 0.01 and ^###^*p* < 0.001 mean significant difference between vehicle and the cisplatin-treated group (CP).

## Data Availability

The data used to support the findings of this study are available from the corresponding author upon request.
